# The mitochondrial genome of *Lichtwardtia dentalis* Zhang, Masunaga et Yang, 2009 (Diptera: Dolichopodidae)

**DOI:** 10.1080/23802359.2021.1948369

**Published:** 2021-07-06

**Authors:** Chen Lin, Ding Yang

**Affiliations:** a Institute of Life Science and Technology, Inner Mongolia Normal University, Hohhot, PR China; b College of Plant Protection, China Agricultural University, Beijing, PR China

**Keywords:** Mitochondrial genome, Dolichopodidae, *Lichtwardtia dentalis*, phylogenetics

## Abstract

The long-legged fly *Lichtwardtia dentalis* Zhang, Masunaga et Yang, 2009 belongs to the subfamily Dolichopodinae of Dolichopodidae. The newly sequenced mitogenome of *L. dentalis* is a new representative of the subfamily. The nearly complete mitogenome is 15,124 bp in length, consisting of 13 protein-coding genes (PCGs), 2 ribosomal RNA genes (rRNAs), and 22 transfer RNA genes (tRNAs). All genes have similar locations and encoding directions with that of other published mitogenomes of Dolichopodidae. The nucleotide composition biases toward A and T with the overall A + T % is 73.9%. All protein-coding genes initiate with standard start codon ATN except *COI* and *NAD1*, and TAA/TAG are conventionally used as stop codons. All tRNAs, ranging from 62 to 71 bp, have a clover-leaf structure. Based on the result of the phylogenetic analysis, Dolichopodidae and Empididae were monophyletic, and the relationships among subfamilies of Dolichopodidae were Diaphorinae + (Peloropeodinae + (Xanthochlorinae + (Medeterinae + Dolichopodinae))). The monophyly of the subfamily Dolichopodinae and the sister relationship between *Dolichopus* and *Lichtwardtia* were also strongly supported.

## Introduction

The subfamily Dolichopodinae is the largest subfamily of the subfamily Dolichopodidae with 1690 known species worldwide (Yang et al. 2006). The genus *Lichtwardtia* is a small group of Dolichopodinae with 20 known species from the world, and three species are known to occur in China (Yang et al. 2006; Yang et al. 2011). Species of *Lichtwardtia* primarily distribute in the Oriental, Australian, and Afrotropical Regions. Adults of *Lichtwardtia* are usually found on the stones and plants near the streams (Yang et al. 2011).

The specimens of *Lichtwardtia dentalis* Zhang, Masunaga *et* Yang, 2009 used for this study were collected by Ding Yang from Yangmeikeng in Shenzhen city of Guangdong province (22°32′N, 114°33′E) on 30 October 2020. These specimens were identified by Prof. Ding Yang based on the combination of the following characters: proboscis dark yellow with yellow palpus; legs yellow except fore and hind coxae entirely yellow, mid coxa mostly yellow with one black outer stripe; hind tarsomere 1 with 1 dorsal bristle and 3 ventral bristles; cercus short, nearly triangular with distinct marginal bristles; hypandrium somewhat acute apically; aedeagus with black denticles apically (Zhang et al. 2009; Yang et al. 2011). Specimens are preserved in 95% ethanol and stored at −20 °C refrigerator in the Entomological Museum of China Agricultural University (Liang Wang, 1352659341@qq.com) under the voucher number CAU-YDLCEMPI-Lide-3. Dolichopodidae is a large, cosmopolitan family with 17 subfamilies. Phylogenetic relationships within the Dolichopodidae are not yet satisfactorily resolved (Sinclair and Cumming 2006; Moulton and Wiegmann 2007). The phylogenetic study based on molecular data can provide a new perspective for studying the evolution and systematics of Dolichopodidae. The mitochondrial DNA is considered as an effective molecular marker and commonly used to investigate the population structure, phylogeography, and phylogenetic analyses of insects (Ma et al. 2012; Zhang et al. 2013), so we added other mitogenomes from Dolichopodidae for further multiple phylogenetic analysis.

The total genomic DNA was extracted from adult’s whole body (except head and wings) using the DNeasy DNA Extraction Kit (TIANGEN, Beijing, China) and stored at −20 °C refrigerator. DNA samples were pooled for next-generation sequencing library construction following the method of Gillett et al. (2014). The library building and sequencing were conducted by BIONONA CO., LTD on an Illumina HiSeq 2500. Rough read data were trimmed and cropped in Trimmomatic version 0.30 with the default setting (Bolger et al. 2014). 4GB of high-quality reads were used to assemble mitogenomes with the *de novo* assembler IDBA-UD (Peng et al. 2012). Theposition of all *tRNA* genes were confirmed using tRNAscanSE version 2.0 (Lowe and Chan 2016). The annotation was conducted using MITOS version 2 WebServer (Bernt et al. 2013), followed by manual adjustments.

The nearly complete mitochondrial genome of *L. dentalis* is 15,124 bp in length, with an A + T content of 73.9% (GenBank accession number: MW526994). It contains all 37 typical insect mitogenomic genes, but we could not get the complete control region sequence because it is particularly difficult to characterize considering the variable sequence and high AT contents of the control region (Wang et al. 2016; Hou et al. 2019; Qilemoge et al. 2020; Wang et al. 2021). All genes have similar locations with that of other published Dolichopodidae mitogenomes. Among the protein-coding genes, six genes took the start codon of ATG (*COII*, *COIII*, *ATP6*, *NAD4*, *NAD4L,* and *CYTB*), three genes used ATT (*NAD2*, *ATP8,* and *NAD5*) as start codon, two genes used ATC (*NAD3*, *NAD6*), while *COI* gene and *NAD1* gene used TCG and TTG, respectively. All the protein-coding genes used the conventional stop codons (TAG for *NAD1*, *NAD3*, *NAD4*, and *CYTB*, TAA for the rest). The length of tRNA genes ranges from 62 to 71 bp. All tRNA genes can be folded into the typical clover-leaf secondary structure. The lrRNA is 1319 bp in length with 79.0% A + T content, and the srRNA is 793 bp with 76.3% A + T content.

**Figure 1.**
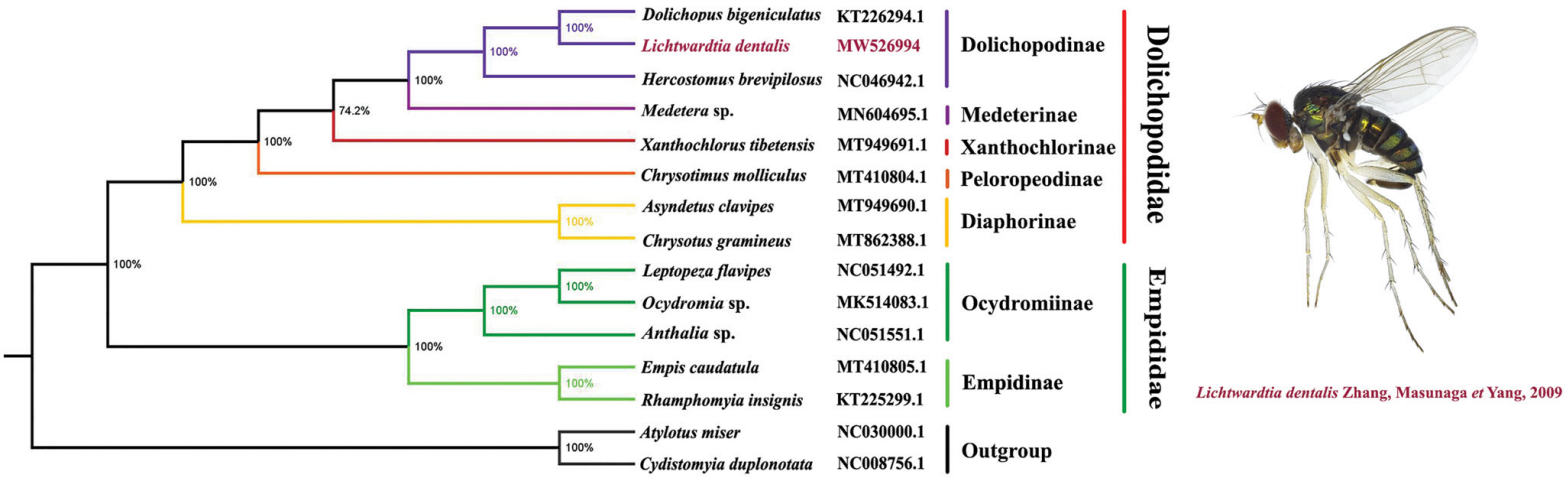
Bayesian phylogenetic tree based on 13 PCGs of 15 Empidoidea species. Genbank accession numbers of all sequences used in the phylogenetic tree have been included in the figure and corresponding to the names of all species. Red words indicated newly sequenced data in this study.

To further validate the mitogenome of *L. dentalis*, the phylogenetic analysis was performed using Bayesian inference (BI) under GTR model in MrBayes version 3.2.7a (Ronquist et al. 2012) based on the concatenated dataset (using all PCGs) of mitogenomes of *L. dentalis* and other 14 taxa that were retrieved from GenBank (Figure 1). The phylogenetic relationship within Dolichopodidae inferred with the Bayesian analysis was stable and clear: Diaphorinae + (Peloropeodinae + (Xanthochlorinae + (Medeterinae + Dolichopodinae))). The monophyly of the subfamily Dolichopodinae and the sister relationship between *Dolichopus* and *Lichtwardtia* were strongly supported. This result also suggested that Dolichopodidae and Empididae are monophyletic, which is consistent with the phylogenetic result of the previous research (Wang et al. 2016).

## Data Availability

The genome sequence data that support the findings of this study are openly available in GenBank of NCBI at [https://www.ncbi.nlm.nih.gov] (https://www.ncbi.nlm.nih.gov/nuccore/MW526994) under the accession No. MW526994. The associated BioProject, SRA, and Bio-Sample numbers are PRJNA722096, SRR14278435, and SAMN18744522, respectively.

## References

[CIT0001] Bernt M, Donath A, Jühling F, Externbrink F, Florentz C, Fritzsch G, Pütz J, Middendorf M, Stadler PF. 2013. MITOS: improved *de novo* metazoan mitochondrial genome annotation. Mol Phylogenet Evol. 69(2):313–319. 22982435 10.1016/j.ympev.2012.08.023

[CIT0002] Bolger AM, Lohse M, Usadel B. 2014. Trimmomatic: a flexible trimmer for Illumina sequence data. Bioinformatics. 30(15):2114–2120. 24695404 10.1093/bioinformatics/btu170 PMC4103590

[CIT0003] Gillett CPDT, Crampton-Platt A, Timmermans MJTN, Jordal BH, Emerson BC, Vogler AP. 2014. Bulk *de novo* mitogenome assembly from pooled total DNA elucidates the phylogeny of weevils (Coleoptera: Curculionoidea)). Mol Biol Evol. 31(8):2223–2237. 24803639 10.1093/molbev/msu154 PMC4104315

[CIT0004] Hou P, Qilemoge , Li X, Yang D. 2019. The mitochondrial genome of *Syntormon pallipes* (Diptera: Dolichopodidae). Mitochondrial DNA Part B. 4(1):1605–1606.

[CIT0005] Lowe TM, Chan PP. 2016. tRNAscan-SE on-line: integrating search and context for analysis of transfer RNA genes. Nucleic Acids Res. 44(W1):W54–W57. 27174935 10.1093/nar/gkw413 PMC4987944

[CIT0006] Ma C, Yang P, Jiang F, Chapuis MP, Shali Y, Sword GA, Kang L. 2012. Mitochondrial genomes reveal the global phylogeography and dispersal routes of the migratory locust. Mol Ecol. 21(17):4344–4358. 22738353 10.1111/j.1365-294X.2012.05684.x

[CIT0007] Moulton JK, Wiegmann BM. 2007. The phylogenetic relationships of flies in the superfamily Empidoidea (Insecta: Diptera). Mol Phylogenet Evol. 43(3):701–713. 17468014 10.1016/j.ympev.2007.02.029

[CIT0008] Peng Y, Leung HCM, Yiu SM, Chin FYL. 2012. IDBA-UD: a *de novo* assembler for single-cell and metagenomic sequencing data with highly uneven depth. Bioinformatics. 28(11):1420–1428. 22495754 10.1093/bioinformatics/bts174

[CIT0009] Qilemoge , Lin C, Fatima N, Yang D. 2020. The mitochondrial genome of *Hercostomus brevipilosus* (Diptera: Dolichopodidae). Mitochondrial DNA Part B. 5(1):685–686. 33366703 10.1080/23802359.2020.1714497 PMC7748520

[CIT0010] Ronquist F, Teslenko M, Van D, Ayres D, Darling A, Höhna S, Larget B, Liu L, Suchard M, Huelsenbeck JP. 2012. MrBayes 3.2: efficient Bayesian phylogenetic inference and model choice across a large model space. Syst Biol. 61(3):539–542. 22357727 10.1093/sysbio/sys029 PMC3329765

[CIT0011] Sinclair BJ, Cumming JM. 2006. The morphology, higher-level phylogeny and classification of the Empidoidea (Diptera). Zootaxa. 1180(1):1–172.

[CIT0012] Wang J, Ji YT, Zhang LS, Wang MQ. 2021. The mitochondrial genome of *Xanthochlorus tibetensis* (Diptera: Dolichopodidae). Mitochondrial DNA Part B. 6(2):515–516. 33628909 10.1080/23802359.2021.1872444 PMC7889179

[CIT0013] Wang K, Wang Y, Yang D. 2016. The complete mitochondrial genome of a stonefly species, *Togoperla* sp. (Plecoptera: Perlidae). Mitochondrial DNA Part A. 27:1703–1704. 10.3109/19401736.2014.961130 25242178

[CIT0014] Yang D, Zhang LL, Wang MQ, Zhu YJ. 2011. Fauna Sinica Insecta. Vol. 53. Diptera Dolichopodidae. Beijing, China: Science Press.

[CIT0015] Yang D, Zhu YJ, Wang MQ, Zhang LL. 2006. World catalog of Dolichopodidae (Insecta: Diptera). Beijing, China: China Agricultural University Press.

[CIT0016] Zhang HL, Zeng HH, Huang Y, Zheng ZM. 2013. The complete mitochondrial genomes of three grasshoppers, *Asiotmethis zacharjini*, *Filchnerella helanshanensis* and *Pseudotmethis rubimarginis* (Orthoptera: Pamphagidae). Gene. 517(1):89–98. 23291499 10.1016/j.gene.2012.12.080

[CIT0017] Zhang LL, Masunaga K, Yang D. 2009. Species of *Lichtwardtia* from China (Diptera: Dolichopodidae). Trans Am Entomol Soc. 135(1–2):197–203.

